# Delayed tension pneumocephalus and pneumorrhacis after routine cervical spine surgery treated successfully without burr holes

**DOI:** 10.1308/rcsann.2023.0037

**Published:** 2023-06-29

**Authors:** Y Lim, A Dahapute, A Clarke, M Hutton, W Selbi

**Affiliations:** Royal Devon and Exeter NHS Foundation Trust, UK

**Keywords:** Tension pneumocephalus, Complication, Burr hole, Cervical, Spine

## Abstract

Tension pneumocephalus (TP) after spinal surgery is very rare with only a few cases reported in the English literature. Most cases of TP occur rapidly after spinal surgery. Traditionally, TP is managed using burr holes to relieve intracranial pressure. However, our case highlights a rare delayed presentation of TP and pneumorrhacis 1 month after routine cervical spine surgery. It is to our knowledge the first case of TP after spinal surgery to be treated using dural repair and supportive care. A 75-year-old woman presented with TP after having routine cervical decompression and stabilisation for cervical myelopathy. She re-presented 1 month after her initial operation with a leaking wound and altered mental status, which deteriorated rapidly shortly after admission. This, in combination with her radiographic features, influenced the decision to explore her surgical wound emergently. She made a full recovery and was discharged after 2 weeks in hospital. We hope to emphasise the need for a high index of suspicion for cerebrospinal fluid leaks and the low threshold to return to theatre to repair a potential dural defect, as well as illustrate that TP after spinal surgery can be treated successfully without burr holes.

## Background

Pneumocephalus is the presence of air in between the leptomeninges (epidural, subdural and/or subarachnoid spaces) or in the brain parenchyma, its vasculature or ventricular system.^1^

Most collections are small, but when they become large enough to exert pressure onto the brain parenchyma, it is known as tension pneumocephalus (TP). TP is caused mostly by trauma to the cranium, but may also be due to infections, tumours and spinal anaesthesia.^1^ Spinal surgery has been reported to be a cause, but TP after spinal surgery is very rare.

We present a case of a 75-year-old woman who presented a month after her cervical spine operation with TP, requiring emergency intervention.

## Case history

**Figure 1:**
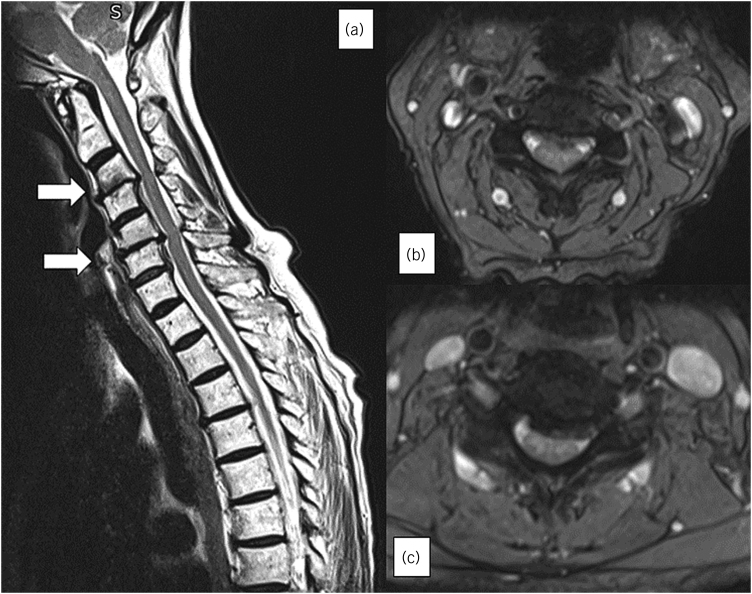
(a) Canal stenosis at levels C3/4 and C5/6. (b, c) Axial MRI T2 cuts of the levels respectively. MRI = magnetic resonance imaging

A 75-year-old female patient initially underwent elective posterior decompression at C3/4 and C5/6 for cervical myelopathy (Figure 1). Intraoperatively, a dural tear with cerebrospinal fluid (CSF) leak was identified and managed with fibrin sealant (Tisseel^®^) and the wound was closed in layers. She recovered well from surgery and was discharged on day 1.

**Figure 2:**
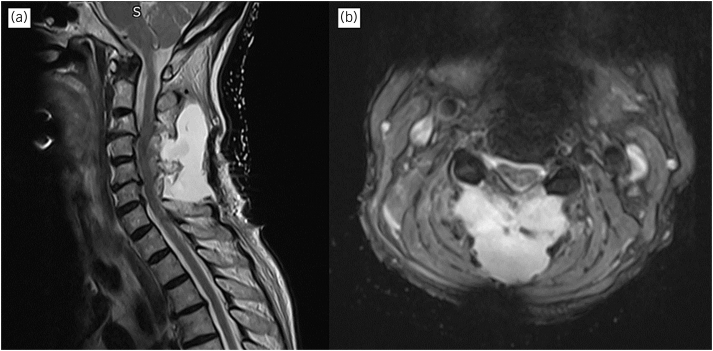
(a) T2 Sagittal MRI at C4-6 illustrating a simple fluid collection. The appearances are suggestive of contained CSF leak. (b) Transverse T2 shows the close relationship of the collection with the spine. CSF = cerebrospinal fluid; MRI = magnetic resonance imaging

She re-attended on day 6 with worsening headaches and burning pains in her arms. Neurological examination was reassuring and the wound was healthy, and had no leak. A magnetic resonance imaging scan (MRI) showed satisfactory decompression but also showed subcutaneous fluid collection (Figure 2). The collection was felt to be a postoperative change and, after discussion with the patient, she opted for nonoperative management and her symptoms gradually resolved.

**Figure 3:**
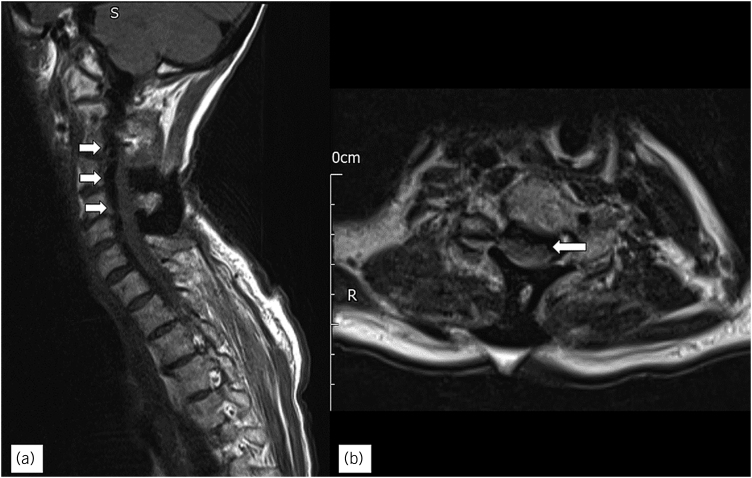
(a) T2 Sagittal MRI showing the ruptured collection communicating with the skin at the C4/5. Gas is tracking into the spinal canal (pneumorrhachis), and intracranially. (b) Communication of air with the spine (arrow). MRI = magnetic resonance imaging

**Figure 4:**
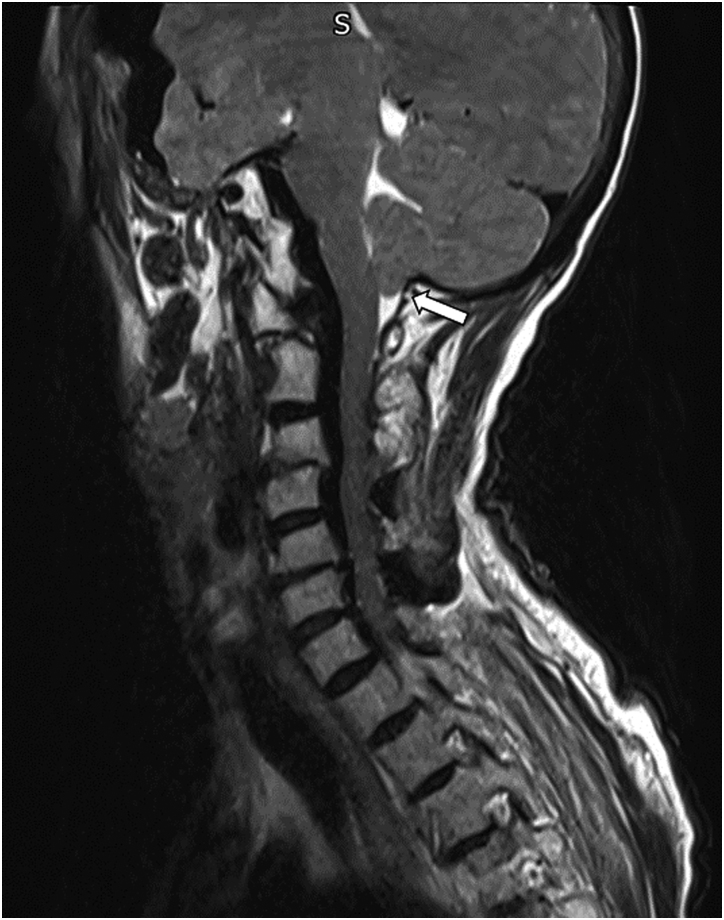
Tonsillar descent through the foramen magnum (arrow)

She presented 3 weeks later with a wound discharging clear fluid, with agitation and confusion. She did not report fever or photophobia, and the wound was not cellulitic. Blood tests showed leukocytosis at 20×10^9^/l and C reactive protein at 4mg/dl. An urgent MRI was performed showing extensive pneumocephalus, pneumorrhacis (Figure 3) and also impending brain herniation (Figure 4).

She was discussed with our regional neurosurgery unit and was advised for conservative management at this stage. She was admitted to the high dependency unit for neurological observations, and intravenous meropenem was started for suspected meningitis. She, unfortunately, dropped her Glasgow coma scale from 14 to 6 after 2h, without clinical seizures. Emergency computed tomography (CT) was performed, showing the pathognomonic Mount Fuji sign for TP.

The patient was transferred emergently to the operating theatre for cervical wound washout and dural repair. The patient was positioned prone, in the Mayfield frame, and a small dural defect was repaired using 6.0 Prolene and the dura was covered with fibrin sealant and the wound was closed without a drain.

**Figure 5:**
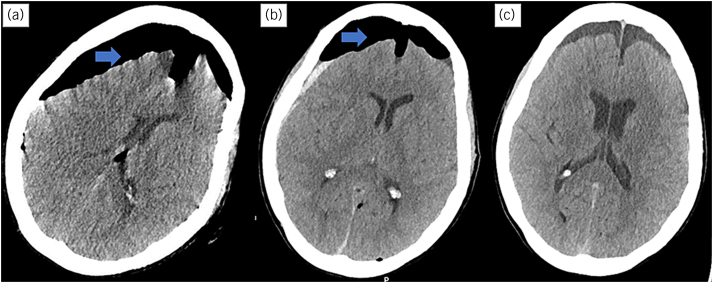
(a) On admission. (b) Day 1 postoperation. (c) Day 9 postoperation.

She was moved to the intensive care unit (ITU) and extubated in the morning. Her CT head scan was repeated on day 1, which showed some resolution of TP. She continued to improve and was stepped down to the ward for neurorehabilitation on day 3. Another CT scan was done on day 9, which confirmed the resolution of TP. Figure 5 illustrates the evolution of her CT head findings.

She was discharged from the hospital on day 14. She was followed up at 4 months in clinic with full recovery and was discharged from our spinal service.

## Discussion

The development of pneumocephalus after spine surgery is attributed to the inverted bottle theory, where the CSF leak reduces intracranial pressure and promotes the entry of air to equalise pressures.^1^ Patients may initially experience headaches, nausea, dizziness and vomiting. As the pressure increases, confusion, seizures and focal neurological deficits such as cranial nerve palsies, sensory or motor changes in limbs may occur, signifying TP, a neurosurgical emergency.^1^

Based on our literature review, five other cases of TP after spinal surgery have been reported in English to date (Table 1), two of which were treated with burr holes and three using conservative treatment, i.e. bed rest and oxygen therapy, which have also been successful (Table 1). To our knowledge, TP with neurological compromise after spinal surgery has not been treated using dural repair only. However, dural repair alone has been employed in a similar TP case without neurological compromise, as well as in a nontension case (Table 1).

**Table 1:** Summary of case reports of pneumocephalus following spinal surgery

	Age	Sex	Surgery	Dural tear	Drain	Symptoms	TP present?	Time of onset	Treatment	Time to extubation
Present case	75	F	C3/4+C5/6 posterior decompression and stabilisation	+	–	Headache, radiating pain in arms, wound discharge, coma	+	3 weeks	Dural repair	12h
Maupin *et al*^2^	66	M	C3–C6 laminoplasty with bilateral foraminotomies	+	–	Altered mental status, decreased mentation, lethargy, wound discharge	+	32 days	Bedside burr hole, dural repair using sutures, tissue patch and glue.	2 days
Gokmen *et al*^3^	56	M	C7-8 drainage of epidural abscess by anterior approach	+	–	Bilateral leg weakness, decreasing consciousness	+	4 days	Burr hole and drain insertion	<24h
Turgut and Akyüz^4^	47	M	L4 Hemi-laminectomy and discectomy	+	+	Low pressure retro-orbital headache, and photophobia	–	2 days	Conservative	Not intubated
Ayberk *et al*^5^	55	F	L4 total laminectomy, and partial laminectomies at L3 and L5 with interbody cage and stabilisation	–	+	Headache, nausea, epistaxis.	+	2 days	Conservative	Not intubated
Kundangar *et al*^6^	20	M	Thoracic spine – removal of metalwork for correction of scoliosis	+	+	Headache, then fever	+	3 days	Conservative	Not intubated
Gupta *et al*^7^	70	M	L4-S1 decompression laminoforaminotomy	–	–	Wound discharge, headache	+	1 week	Wound debridement and primary watertight closure of layers	Not intubated
Yun *et al*^8^	59	M	L4 Subtotal laminectomy and L5 Total laminectomy with pedicle screw fixation	+	–	Progressive headache, dizziness, and low mood	–	1 day	Conservative	Not intubated
Ozturk *et al*^9^	23	F	Thoracolumbar pedicle screw fixation for scoliosis	+	+	Headache, nausea, and coma	–	3h	Dural repair	Not intubated
Nowak *et al*^10^	12	F	T2–T4 pedicle screw fixation	+	+	Increasing drowsiness (thought to be anaesthetic initially), and aphasia.	–	6h	Burr hole. Dural repair was done 2 weeks after.	Not intubated
Dhamija *et al*^11^	63	F	Revision L3/4 and L4/5 decompression and stabilisation	+	–	Headache, confusion, restlessness, and mild pyrexia (37.9°C)	–	<24h	Conservative	Not intubated
Karavelioglu *et al*^12^	56	M	Hemilaminectomy at L3, L4, L5 and stabilisation	–	+	Severe headache, nausea, dizziness	–	1 day	Conservative	Not intubated
Gauthe *et al*^13^	69	M	L2–L4 Decompression, fusion and stabilisation	–	+	Severe back pain, tonic-clonic seizure	–	1 day	Conservative	Not intubated

Our patient recovered and was extubated more quickly than has been reported in a case of conservative management,^5^ which is similar to burr hole therapy alone.^3^ Our patient also recovered quicker compared with cases treated with burr holes and dural repair.^2^ It should be highlighted that it is difficult to draw definitive conclusions due to the heterogeneity of patient groups as well as small sample size, but – at least in our patient – dural repair with supportive care had similar, if not better, outcomes than reported cases that have been treated with burr holes.

Similar cases of delayed presentation and leaking wounds have also been reported by Gupta *et al* and Maupin *et al*, both presenting 1 month after their operations.^2,7^ Maupin *et al* reported a clean wound that had sutures removed 2 days prior to re-presentation;^2^ Gupta *et al* did not comment on the wound prior to re-presentation.^7^ In our case, we suggest that a delayed presentation of TP is still possible despite a superficially healed wound.

Incidental durotomy or dural tear is a not uncommon complication of spine surgery, with rates ranging between 1.8% and 17.4%.^14^ It is more likely to occur in operations around the thoracic and lumbar spine, and is relatively rare in operations of the cervical spine, with an estimated incidence of 1%.^15^ Although most durotomies are asymptomatic, having a dural tear is a common risk factor in the development of pneumocephalus, which increases the risk of CSF leak, and ultimately the risk of developing TP.^16^

If CSF leak is suspected postoperatively, an MRI scan of the spine is the imaging modality of choice to investigate but there should be a low threshold to do CT head as this will clarify whether there is pneumocephalus or not. If a collection is present (over operation site), then this should be assumed to be CSF until proven otherwise and should raise concerns over an unrecognised or inadequately repaired dural defect. The risks and benefits of exploring the surgical wound should then be carefully explained to the patient.

## Conclusion

Our case highlights a rare complication of routine spinal surgery and the successful outcome of treating TP (postspinal CSF leak) without burr holes. Current guidelines do not exist due to the nature of the problem, but we hope reporting cases like these may aid in recognition and clinical decision, as well as the potential development of treatment guidelines.

## Consent for publication

A consent form has been signed by the patient. The original of the signed form is held by the institution and can be made available to the editors upon request.

## References

[1] Das JM, Bajaj J. *Pneumocephalus. StatPearls*. Treasure Island (FL): StatPearls Publishing; 2022. https://www.ncbi.nlm.nih.gov/books/NBK535412/ (cited June 2024).

[2] Maupin J, Burrow Z, Shirazi C, Vallurupalli S. Tension pneumocephalus after cervical spine surgery: a case report with review of the literature. *J Neurol Surg Rep* 2018; **79**: e88–e92. 30510888 10.1055/s-0038-1676298 PMC6269234

[3] Gokmen IE, Keskin S, Kıresi D, Erdoğan H. Mount Fuji sign following surgical drainage of spinal epidural abscess: figure 1. *QJM* 2015; **108**: 835–836. 25862771 10.1093/qjmed/hcv078

[4] Turgut M, Akyüz O. Symptomatic tension pneumocephalus: an unusual post-operative complication of posterior spinal surgery. *J Clin Neurosci* 2007; **14**: 666–668. 17532503 10.1016/j.jocn.2006.02.021

[5] Ayberk G, Yaman ME, Ozveren MF. Symptomatic spontaneous pneumocephalus after spinal fusion for spondylolisthesis. *J Clin Neurosci* 2010; **17**: 934–936. 20400320 10.1016/j.jocn.2009.10.033

[6] Kundangar RS, Bhat SN, Mohanty SP. Tension pneumocephalus following an implant removal from spine. *BMJ Case Rep* 2021; **14**: e239694. 10.1136/bcr-2020-239694 PMC7919551 33627348

[7] Gupta M, Kumar Varma KK, Singh Chhabra H. A rare case of concomitant pneumocephalus and pneumorachis after lumbar spine surgery with late presenting dural leak. *Spinal Cord Ser Cases* 2019; **5**. 10.1038/s41394-019-0235-3 PMC6821870 31700684

[8] Yun JH, Kim YJ, Yoo DS *et al.* Diffuse pneumocephalus: a rare complication of spinal surgery. *J Korean Neurosurg Soc* 2010; **48**: 288–290. 21082062 10.3340/jkns.2010.48.3.288 PMC2966736

[9] Ozturk E, Kantarci M, Karaman K *et al.* Diffuse pneumocephalus associated with infratentorial and supratentorial hemorrhages as a complication of spinal surgery. *Acta Radiol* 2006; **47**: 497–500. 16796314 10.1080/02841850600644766

[10] Nowak R, Maliszewski M, Krawczyk L. Intracranial subdural hematoma and pneumocephalus after spinal instrumentation of myelodysplastic scoliosis. *J Pediatr Orthop B* 2011; **20**: 41–45. 20829719 10.1097/BPB.0b013e32833f33d1

[11] Dhamija B, Saxena A. Pneumocephalus - a possible cause of post-spinal surgery confusion. *J R Soc Med* 2011; **104**: 81–83. 21282798 10.1258/jrsm.2010.100311 PMC3031647

[12] Karavelioglu E, Eser O, Haktanir A. Pneumocephalus and pneumorrhachis after spinal surgery: case report and review of the literature. *Neurol Med Chir (Tokyo)* 2014; **54**: 405–407. 24305016 10.2176/nmc.cr2013-0118 PMC4533435

[13] Gauthé R, Latrobe C, Damade C *et al.* Symptomatic compressive pneumocephalus following lumbar decompression surgery. *Orthop Traumatol: Surg Res* 2016; **102**: 251–253. 26796946 10.1016/j.otsr.2015.12.006

[14] Khan MH, Rihn J, Steele G *et al.* Postoperative management protocol for incidental dural tears during degenerative lumbar spine surgery. *Spine* 2006; **31**: 2609–2613. 17047553 10.1097/01.brs.0000241066.55849.41

[15] O'Neill KR, Neuman BJ, Peters C, Riew KD. Risk factors for dural tears in the cervical spine. *Spine* 2014; **39**: E1015–E1020. 24859583 10.1097/BRS.0000000000000416

[16] Greenberg MS. *Handbook of Neurosurgery.* Stuttgart, Germany: Thieme Medical Publishers; 2019.

